# Supplementary material for: Fitting and comparison of calcium-calmodulin kinetic schemes to a common data set using non-linear mixed effects modelling
Source: PLoS One. 2025 Feb 7;20(2):e0318646. doi: 10.1371/journal.pone.0318646 (PMC11805441; doi:10.1371/journal.pone.0318646)
Supplement: S1 Appendix — (PDF) [file pone.0318646.s001.pdf]

S1 Appendix.

Subset of Data used and its splitting into training, validation and testing data sets

The subset of data that we use from [1] contains 94 recorded time series across 7 groups of initial conditions with varying numbers of time series between the initial conditions. Each time series contains data on fluorescence change divided by basal fluorescence $\Delta F/F_0$. Each time series is a record of either 35.204 ms (for group A) or 39.604 ms (all other groups) of $\Delta F/F_0$ after laser uncaging. Each time series is made up of either 258 (group A) or 260 (all other groups) points that were sampled unevenly – most points were focused in the first 4ms of the time series. The first recorded time point after laser uncaging was at 0.160 ms.

From the full 7 groups we removed two time series from group C because the data them were numerically identical but showed as having two different Pockels cell density values. This left 92 time series for us to use. We split the 92 time series into training, validation and test data sets. The training data set consisted of 7 randomly selected (without replacement) samples from each of the different experimental conditions, leaving 45 time series. The validation data set consisted of time series taken from the remaining 47 by randomly selecting 2–4 time series (without replacement) per experimental condition (due to imbalance in the group sizes). The remaining data was left for final model testing. We repeated this splitting procedure for 20 different seeds in order to counteract the small data set size.

We sub-sampled the first 2.424ms of each time-series due to heavy over-sampling of this time period. As shown in [2], the larger the number of points in a time series used when fitting parameters of dynamical systems, the more complex the loss surface. Therefore, sub-sampling was done to improve the gradient-based optimization done by `Pumas.jl`. Namely, the original set of points $\{d_i\}_{i=1}^{201}$ we uniformly sub-sampled the points to $\{d_{1+20i}\}_{i=0}^{10}$. The comparison of original and sub-sampled data is shown in **??** below.

References

1. Faas GC, Raghavachari S, Lisman JE, Mody I. Calmodulin as a direct detector of Ca2+ signals. Nature Neuroscience. 2011;14:301–304. doi:10.1038/nn.2746.

2. Ribeiro AH, Tiels K, Umenberger J, Schön TB, Aguirre LA. On the smoothness of nonlinear system identification. Automatica. 2020;121. doi:10.1016/j.automatica.2020.109158.
