# Supplementary material for: Fitting and comparison of calcium-calmodulin kinetic schemes to a common data set using non-linear mixed effects modelling
Source: PLoS One. 2025 Feb 7;20(2):e0318646. doi: 10.1371/journal.pone.0318646 (PMC11805441; doi:10.1371/journal.pone.0318646)
Supplement: S2 Appendix — (PDF) [file pone.0318646.s002.pdf]

S2 Appendix.

Concentrations of species of different groups of solutions used in Faas et al. data

Table below shows the initial concentrations for all groups. There are some differences from those provided in the supplemental information in [1] due to personal communications.

	**A**	**B**	**C**	**D**	**E**	**F**	**G**
DMn	5.56mM	3.64mM	3.64mM	3.64mM	3.64mM	3.64mM	3.64mM
OGB-5N	50µM	100µM	100µM	100µM	100µM	100µM	100µM
${Ca^{2+}}_{Free}$	1.88µM	1.10µM	0.73µM	0.255µM	0.41µM	399µM	394µM
CaM	123µM	143µM	72µM	187µM	140µM	94µM	47µM

Table 1. Initial conditions for the 7 experimental groups from [1].

References

1. Faas GC, Raghavachari S, Lisman JE, Mody I. Calmodulin as a direct detector of Ca2+ signals. Nature Neuroscience. 2011;14:301–304. doi:10.1038/nn.2746.
