# Supplementary material for: Fitting and comparison of calcium-calmodulin kinetic schemes to a common data set using non-linear mixed effects modelling
Source: PLoS One. 2025 Feb 7;20(2):e0318646. doi: 10.1371/journal.pone.0318646 (PMC11805441; doi:10.1371/journal.pone.0318646)
Supplement: S3 Appendix — (PDF) [file pone.0318646.s003.pdf]

S4 Appendix.

Derivation of steady-state reaction rate constants for Scheme 4

Since we do not need to track the full CaM species (since it does not bind anything downstream) we consider lobes individually, namely CaM0, CaM1x, CaM2x where x can be either the N or C lobe. This results in a three dimensional ODE system with 8 reaction rate constants (4 per lobe) k_i^x, i ∈ (1, 2, 3, 4), x ∈ (C, N). The resulting ODE system for a single lobe is

$$\frac{d[CaM0]}{dt} = -k_1^x[CaM0][Ca] + k_2^x[CaM1x]$$
$$\frac{d[CaM1x]}{dt} = k_1^x[CaM0][Ca] - k_2^x[CaM1x] - k_3^x[CaM1x][Ca] + k_4^x[CaM2x]$$
$$\frac{d[CaM2x]}{dt} = k_3^x[CaM1x][Ca] - k_4^x[CaM2x]$$

The quasi-steady state approximation means $\frac{d[CaM1x]}{dt} = 0$, therefore we can express $[CaM1x]$ via other terms

$$0 = k_1^x[CaM0][Ca] - k_2^x[CaM1x] - k_3^x[CaM1x][Ca] + k_4^x[CaM2x]$$
$$k_2^x[CaM1x] + k_3^x[CaM1x][Ca] = k_1^x[CaM0][Ca] + k_4^x[CaM2x]$$
$$[CaM1x](k_2^x + k_3^x[Ca]) = k_1^x[CaM0][Ca] + k_4^x[CaM2x]$$
$$[CaM1x] = \frac{1}{k_2^x + k_3^x[Ca]}(k_1^x[CaM0][Ca] + k_4^x[CaM2x])$$

and then substitute back into equations for $\frac{d[CaM0]}{dt}$ and $\frac{d[CaM2x]}{dt}$:

$$\frac{d[CaM0]}{dt} = -k_1^x[CaM0][Ca] + \frac{k_2^x}{k_2^x + k_3^x[Ca]}(k_1^x[CaM0][Ca] + k_4^x[CaM2x])$$
$$\frac{d[CaM2x]}{dt} = \frac{k_3^x[Ca]}{k_2^x + k_3^x[Ca]}(k_1^x[CaM0][Ca] + k_4^x[CaM2x]) - k_4^x[CaM2x]$$

which after multiplying through, gathering the terms and some simplification becomes:

$$\frac{d[CaM0]}{dt} = -\frac{k_1^x k_3^x}{k_2^x + k_3^x[Ca]}[CaM0][Ca]^2 + \frac{k_2^x k_4^x}{k_2^x + k_3^x[Ca]}[CaM2x]$$
$$\frac{d[CaM2x]}{dt} = \frac{k_1^x k_3^x}{k_2^x + k_3^x[Ca]}[CaM0][Ca]^2 - \frac{k_2^x k_4^x}{k_2^x + k_3^x[Ca]}[CaM2x]$$

which if we set

$$k_{ss}^{fx}([Ca]) = \frac{k_1^x k_3^x}{k_2^x + k_3^x[Ca]}$$
$$k_{ss}^{bx}([Ca]) = \frac{k_2^x k_4^x}{k_2^x + k_3^x[Ca]}$$

makes the reduced system

$$\frac{d[CaM0]}{dt} = -k_{ss}^{fx}([Ca])[CaM0][Ca]^2 + k_{ss}^{bx}([Ca])[CaM2x]$$
$$\frac{d[CaM2x]}{dt} = k_{ss}^{fx}([Ca])[CaM0][Ca]^2 - k_{ss}^{bx}([Ca])[CaM2x]$$

easy to interpret as a $CaM0 + 2Ca \Longleftrightarrow CaM2x$ with Ca^{2+}-dependent reaction rates for Ca^{2+} binding to lobe x $\in$ (C, N).
