# Supplementary material for: Fitting and comparison of calcium-calmodulin kinetic schemes to a common data set using non-linear mixed effects modelling
Source: PLoS One. 2025 Feb 7;20(2):e0318646. doi: 10.1371/journal.pone.0318646 (PMC11805441; doi:10.1371/journal.pone.0318646)
Supplement: S4 Appendix — (PDF) [file pone.0318646.s004.pdf]

S5 Appendix.

Published reaction rate constants

**Reaction**	$\log_{10}(\mathbf{k_f}\frac{1}{M\times ms})$	$\log_{10}(\mathbf{k_f}\frac{1}{ms})$	$\log_{10}(\mathbf{K_d}M)$
CaM + 2Ca $\Longleftrightarrow$ CaM_2	3.60	-2.10	-5.70
CaM_2 + 2Ca $\Longleftrightarrow$ CaM_4	5.0	0.04	-4.96

Table 1. Original parameters from [1] for Scheme 1 that were used as a basis for [2] reaction rate constants.

**Reaction**	$\log_{10}(\mathbf{k_f}\frac{1}{M\times ms})$	$\log_{10}(\mathbf{k_f}\frac{1}{ms})$	$\log_{10}(\mathbf{K_d}M)$
CaM + 2Ca $\Longleftrightarrow$ CaM_2	4.86	-1.42	-6.00
CaM_2 + Ca $\Longleftrightarrow$ CaM_3	3.56	-2.00	-5.55
CaM_3 + Ca $\Longleftrightarrow$ CaM_4	2.67	-2.01	-4.68

Table 2. Scheme 2 parameters used in [3].

**Reaction**	$\log_{10}(\mathbf{k_f}\frac{1}{M\times ms})$	$\log_{10}(\mathbf{k_f}\frac{1}{ms})$	$\log_{10}(\mathbf{K_d}M)$
CaM + Ca $\Longleftrightarrow$ CaM_1	-	-	-5.10
CaM_1 + Ca $\Longleftrightarrow$ CaM_2	-	-	-5.77
CaM_2 + Ca $\Longleftrightarrow$ CaM_3	-	-	-4.46
CaM_3 + Ca $\Longleftrightarrow$ CaM_4	-	-	-5.05

Table 3. Scheme 3 parameters from [4].

**Reaction**	$\log_{10}(\mathbf{k_f}\frac{1}{M\times ms})$	$\log_{10}(\mathbf{k_f}\frac{1}{ms})$	$\log_{10}(\mathbf{K_d}M)$
CaM + Ca $\Longleftrightarrow$ CaM_{1C}	3.60	-1.40	-5.00
CaM_{1C} + Ca $\Longleftrightarrow$ CaM_{2C}	4.00	-2.03	-6.03
CaM + Ca $\Longleftrightarrow$ CaM_{1N}	5.00	0.40	-4.60
CaM_{1N} + Ca $\Longleftrightarrow$ CaM_{2N}	5.18	-0.12	-5.30

Table 4. Scheme 4 and 5 parameters from [5].

**Reaction**	$\log_{10}(\mathbf{k_f}\frac{1}{M\times ms})$	$\log_{10}(\mathbf{k_f}\frac{1}{ms})$	$\log_{10}(\mathbf{K_d}M)$
CaM + Ca $\Longleftrightarrow$ CaM_{1C}	4.90	0.30	-4.60
CaM_{1C} + Ca $\Longleftrightarrow$ CaM_{2C}	4.40	-2.20	-6.60
CaM + Ca $\Longleftrightarrow$ CaM_{1N}	5.90	2.20	-3.70
CaM_{1N} + Ca $\Longleftrightarrow$ CaM_{2N}	7.50	1.40	-6.10

Table 5. Scheme 5 parameters from [6].

**Reaction**	$\log_{10}(\mathbf{k_f}\frac{1}{M\times ms})$	$\log_{10}(\mathbf{k_f}\frac{1}{ms})$	$\log_{10}(\mathbf{K_d}M)$
$CaM_{0N} + Ca \Longleftrightarrow CaM_{1N}$	5.44	0.96	-4.48
$CaM_{0N} + Ca \Longleftrightarrow CaM_{2N}$	5.44	-1.80	-3.64
$CaM_{1N} + Ca \Longleftrightarrow CaM_{3N}$	5.71	0.24	-5.46
$CaM_{2N} + Ca \Longleftrightarrow CaM_{3N}$	5.70	-0.60	-6.30
$CaM_{0C} + Ca \Longleftrightarrow CaM_{1C}$	5.44	0.70	-4.73
$CaM_{0C} + Ca \Longleftrightarrow CaM_{2C}$	5.44	1.50	-3.94
$CaM_{1C} + Ca \Longleftrightarrow CaM_{3C}$	3.57	-2.85	-6.42
$CaM_{2C} + Ca \Longleftrightarrow CaM_{3C}$	5.07	-2.14	-7.22

Table 6. Scheme 6 parameters from [7].

References

1. Putkey JA, Waxham MN, Gaertner TR, Brewer KJ, Goldsmith M, Kubota Y, et al. Acidic/IQ motif regulator of calmodulin. Journal of Biological Chemistry. 2008;283:1401–1410. doi:10.1074/jbc.M703831200.

2. Kim M, Huang T, Abel T, Blackwell KT. Temporal sensitivity of protein kinase A activation in late-phase long term potentiation. PLoS Computational Biology. 2010;6. doi:10.1371/journal.pcbi.1000691.

3. Bhalla US, Iyengar R. Emergent properties of networks of biological signaling pathways. Science. 1999;283:381–387. doi:10.1126/science.283.5400.381.

4. Shifman JM, Choi MH, Mihalas S, Mayo SL, Kennedy MB. Ca2+/calmodulin-dependent protein kinase II (CaMKII) is activated by calmodulin with two bound calciums. Proceedings of the National Academy of Sciences. 2006;103(38):13968–13973. doi:10.1073/pnas.0606433103.

5. Pepke S, Kinzer-Ursem T, Mihalas S, Kennedy MB. A dynamic model of interactions of Ca2+, calmodulin, and catalytic subunits of Ca2+/calmodulin-dependent protein kinase II. PLoS Computational Biology. 2010;6. doi:10.1371/journal.pcbi.1000675.

6. Faas GC, Raghavachari S, Lisman JE, Mody I. Calmodulin as a direct detector of Ca2+ signals. Nature Neuroscience. 2011;14:301–304. doi:10.1038/nn.2746.

7. Byrne MJ, Putkey JA, Waxham MN, Kubota Y. Dissecting cooperative calmodulin binding to CaM kinase II: A detailed stochastic model. Journal of Computational Neuroscience. 2009;27:621–638. doi:10.1007/s10827-009-0173-3.
