# Supplementary material for: Fitting and comparison of calcium-calmodulin kinetic schemes to a common data set using non-linear mixed effects modelling
Source: PLoS One. 2025 Feb 7;20(2):e0318646. doi: 10.1371/journal.pone.0318646 (PMC11805441; doi:10.1371/journal.pone.0318646)
Supplement: S5 Appendix — (PDF) [file pone.0318646.s005.pdf]

S6 Appendix.

Our Parameter Fits for All Schemes

References

1. Kim M, Huang T, Abel T, Blackwell KT. Temporal sensitivity of protein kinase A activation in late-phase long term potentiation. PLoS Computational Biology. 2010;6. doi:10.1371/journal.pcbi.1000691.

2. Bhalla US, Iyengar R. Emergent properties of networks of biological signaling pathways. Science. 1999;283:381–387. doi:10.1126/science.283.5400.381.

3. Shifman JM, Choi MH, Mihalas S, Mayo SL, Kennedy MB. Ca2+/calmodulin-dependent protein kinase II (CaMKII) is activated by calmodulin with two bound calciums. Proceedings of the National Academy of Sciences. 2006;103(38):13968–13973. doi:10.1073/pnas.0606433103.

4. Pepke S, Kinzer-Ursem T, Mihalas S, Kennedy MB. A dynamic model of interactions of Ca2+, calmodulin, and catalytic subunits of Ca2+/calmodulin-dependent protein kinase II. PLoS Computational Biology. 2010;6. doi:10.1371/journal.pcbi.1000675.

5. Faas GC, Raghavachari S, Lisman JE, Mody I. Calmodulin as a direct detector of Ca2+ signals. Nature Neuroscience. 2011;14:301–304. doi:10.1038/nn.2746.

6. Byrne MJ, Putkey JA, Waxham MN, Kubota Y. Dissecting cooperative calmodulin binding to CaM kinase II: A detailed stochastic model. Journal of Computational Neuroscience. 2009;27:621–638. doi:10.1007/s10827-009-0173-3.

**Seed #**	$\mathbf{k_1}$	$\mathbf{K_{D_1}}$	$\mathbf{k_2}$	$\mathbf{K_{D_2}}$
Seed 1	8.49	-9.0	8.99	-9.0
Seed 2	2.02	-9.0	7.13	-4.99
Seed 3	6.72	-9.0	9.0	-8.98
Seed 4	8.1	-9.0	3.92	-9.0
Seed 5	8.28	-9.0	3.98	-9.0
Seed 6	8.37	-9.0	6.42	-9.0
Seed 7	8.2	-9.0	4.03	-9.0
Seed 8	8.66	-9.0	7.75	-9.0
Seed 9	8.2	-9.0	4.05	-9.0
Seed 10	8.09	-9.0	3.76	-9.0
Seed 11	8.19	-9.0	8.03	-5.54
Seed 13	3.07	-9.0	6.79	-8.92
Seed 14	8.1	-9.0	7.83	-5.79
Seed 15	8.03	-9.0	4.79	-9.0
Seed 16	8.14	-9.0	2.56	-8.94
Seed 17	8.24	-9.0	5.3	-9.0
Seed 18	8.88	-9.0	7.85	-9.0
Seed 19	8.05	-9.0	7.21	-5.16
Seed 20	8.22	-9.0	6.88	-7.17

Table 1. Our reaction rate fits for Scheme 1 for all seeds. Seed 12 was removed due to training failures.

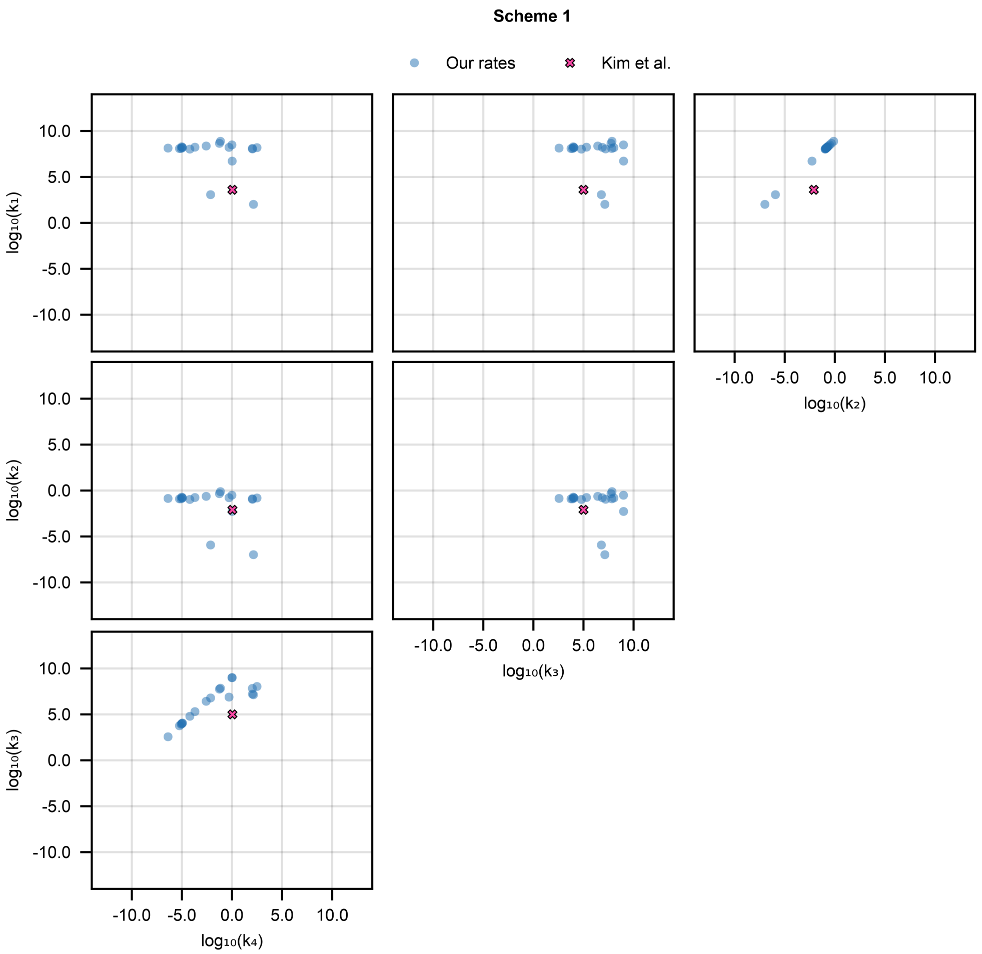

Fig 1. Pair plots for our reaction rate constants for Scheme 1 (blue dots) for all random seeds, along with the published reaction rate constants from Kim et al. [1].

Seed #	k_1	K_{D_1}	k_2	K_{D_2}	k_3	K_{D_3}
Seed 1	8.18	-9.0	2.04	-8.53	4.67	-4.03
Seed 2	2.03	-9.0	6.6	-9.0	4.22	-6.21
Seed 3	6.43	-9.0	9.0	-9.0	4.38	-6.36
Seed 4	8.07	-9.0	2.07	-9.0	4.58	-6.42
Seed 5	8.84	-5.96	2.08	-8.87	4.46	-8.85
Seed 6	8.24	-9.0	2.41	-9.0	4.56	-6.41
Seed 7	6.52	-9.0	6.15	-9.0	4.41	-6.29
Seed 8	9.0	-8.99	7.69	-5.27	2.87	-9.0
Seed 9	8.38	-9.0	2.52	-8.98	2.92	-4.03
Seed 10	6.51	-9.0	6.35	-9.0	4.39	-6.37
Seed 11	6.72	-9.0	6.43	-9.0	4.5	-6.4
Seed 12	7.5	-9.0	5.99	-8.34	5.29	-9.0
Seed 13	6.44	-9.0	6.41	-9.0	4.35	-6.37
Seed 14	6.7	-9.0	7.36	-9.0	4.48	-6.37
Seed 15	6.62	-9.0	6.53	-9.0	4.42	-6.35
Seed 16	8.16	-9.0	2.07	-8.98	4.13	-4.1
Seed 17	7.97	-8.77	6.66	-8.8	8.4	-8.88
Seed 18	6.61	-9.0	6.5	-9.0	4.44	-6.39
Seed 19	8.09	-9.0	2.02	-6.18	8.75	-8.86
Seed 20	6.54	-9.0	6.29	-9.0	4.42	-6.36

Table 2. Our reaction rate fits for Scheme 2 for all seeds.

Seed #	k_1	K_{D_1}	k_2	K_{D_2}	k_3	K_{D_3}	k_4	K_{D_4}
Seed 1	4.28	-6.26	5.81	-4.83	7.67	-3.61	7.91	-5.74
Seed 2	2.1	-3.23	3.97	-9.44	4.38	-6.27	5.4	-5.3
Seed 3	4.2	-6.46	5.47	-5.47	7.68	-2.8	8.53	-6.26
Seed 4	5.94	-4.83	6.0	-5.77	4.66	-6.05	3.17	-4.15
Seed 5	5.96	-7.85	4.21	-6.43	7.9	-3.09	6.94	-6.63
Seed 6	6.77	-4.7	5.78	-6.03	4.36	-5.69	6.78	-4.01
Seed 7	4.29	-6.26	5.67	-4.98	7.55	-4.21	7.37	-5.08
Seed 8	4.32	-6.62	5.23	-4.79	2.07	-3.38	6.36	-6.1
Seed 9	4.22	-6.33	5.54	-5.19	7.53	-3.4	7.84	-5.76
Seed 10	5.76	-5.84	4.5	-5.64	2.36	-6.83	5.51	-2.39
Seed 11	4.28	-6.2	5.83	-4.75	7.67	-3.3	8.2	-6.01
Seed 12	4.27	-6.32	7.71	-3.17	7.08	-6.08	2.18	-4.96
Seed 13	2.01	-6.41	5.64	-5.77	4.6	-6.94	3.28	-3.98
Seed 14	7.18	-3.36	6.97	-7.52	4.41	-5.94	6.87	-4.18
Seed 15	4.35	-6.64	5.31	-4.83	2.11	-3.26	6.53	-6.19
Seed 16	5.46	-5.09	2.59	-8.02	4.36	-6.1	5.53	-4.67
Seed 17	5.6	-7.06	4.21	-6.42	7.71	-3.06	7.05	-6.59
Seed 18	4.12	-6.34	5.59	-4.77	7.59	-3.3	7.92	-6.03
Seed 19	5.38	-4.89	2.74	-8.22	4.37	-6.1	5.57	-4.48
Seed 20	4.32	-6.29	7.73	-3.43	6.9	-5.89	2.29	-4.86

Table 3. Our reaction rate fits for Scheme 3 for all seeds.

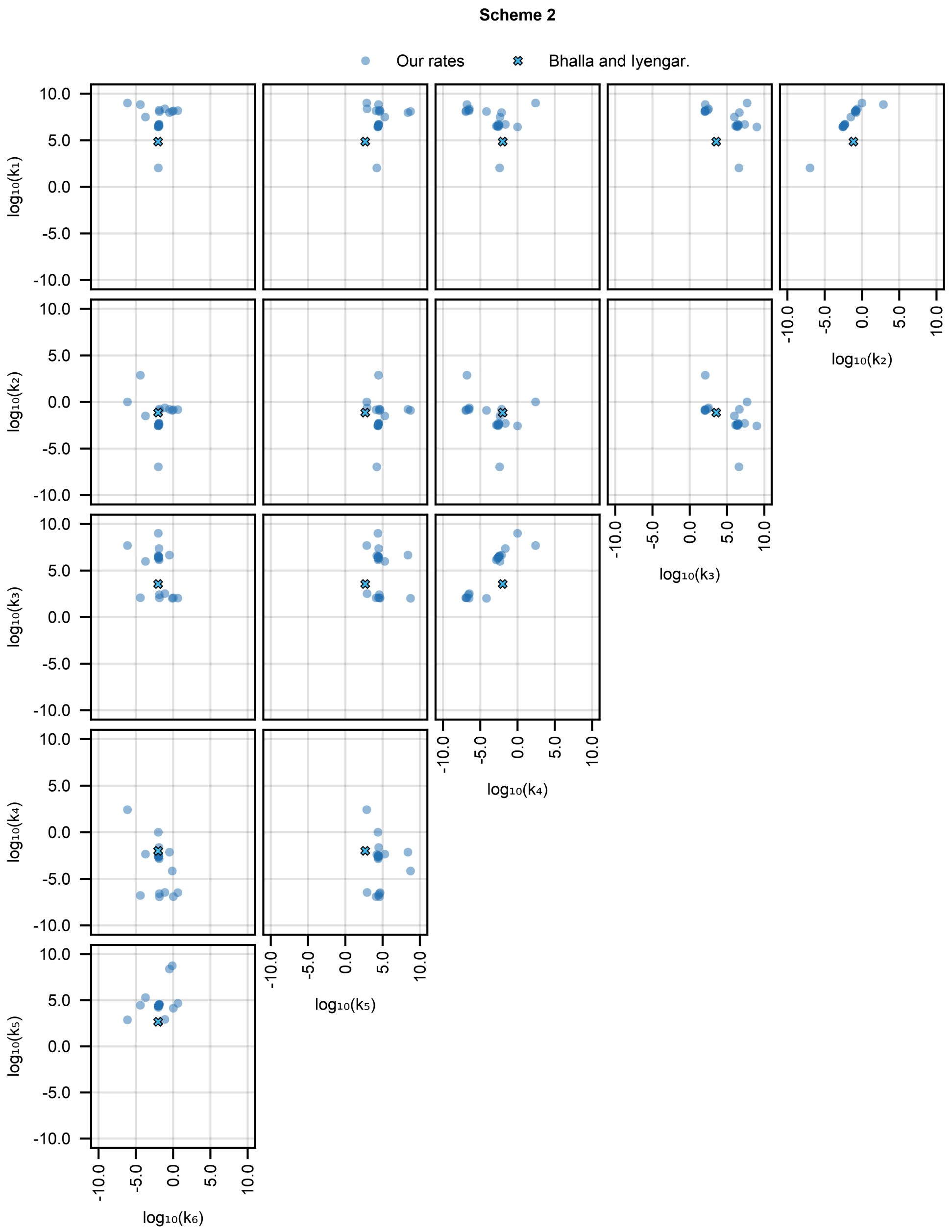

Fig 2. Pair plots for our reaction rate constants for Scheme 2 (blue dots) for all random seeds, along with the published reaction rate constants from Bhalla and Iyengar [2].

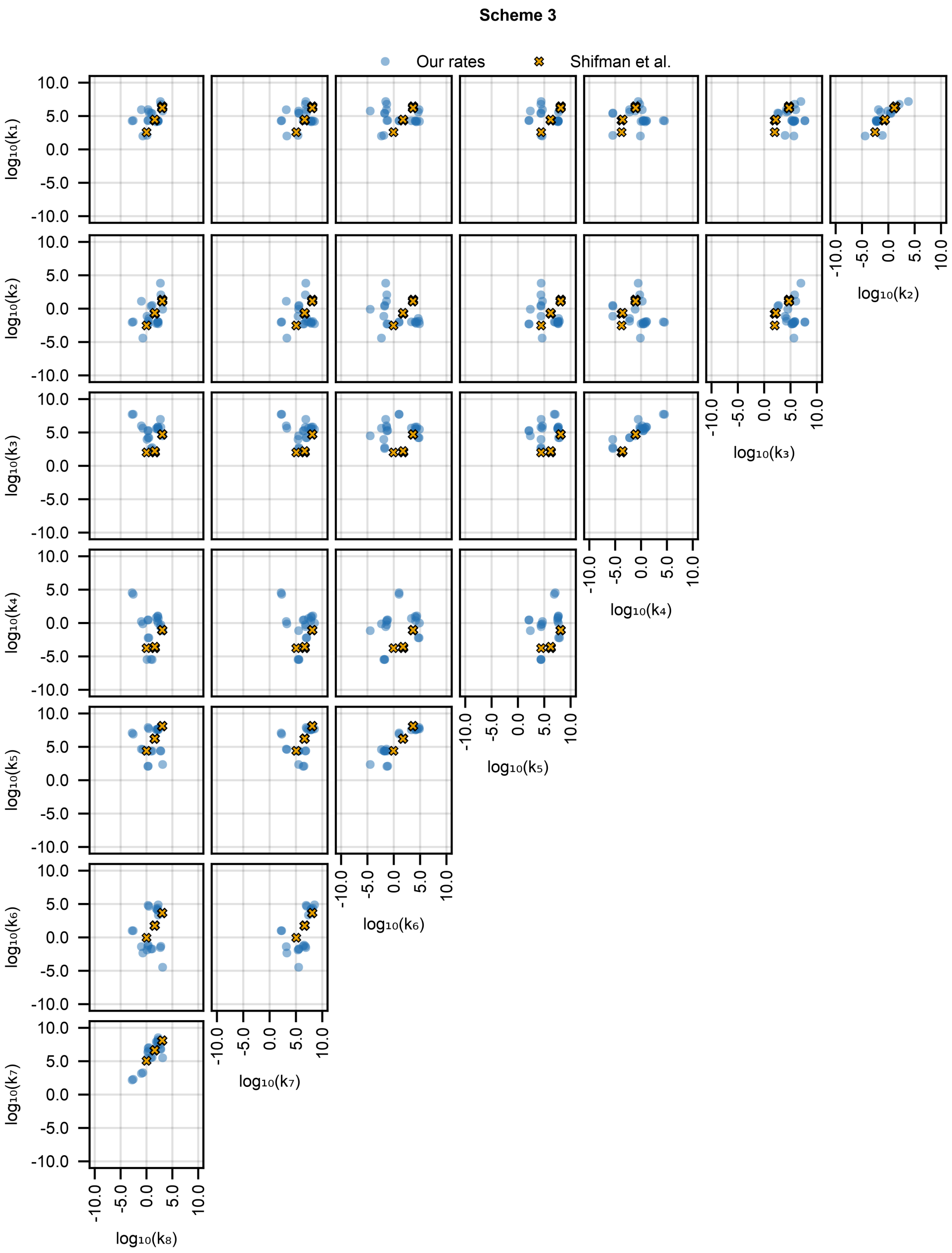

Fig 3. Pair plots for our reaction rate constants for Scheme 3 (blue dots) for all random seeds, along with the published reaction rate constants from Shifman et al. [3].

Seed #	k_5	K_{D_5}	k_7	K_{D_7}	k_1	K_{D_1}	k_3	K_{D_3}
Seed 1	6.52	-4.18	6.4	-5.67	4.38	-5.28	5.5	-6.2
Seed 2	6.16	-4.13	6.3	-5.73	4.35	-5.21	5.26	-6.37
Seed 3	2.1	-4.06	7.51	-4.44	6.9	-5.32	4.02	-7.73
Seed 4	6.68	-4.07	6.31	-5.66	4.35	-5.1	5.19	-6.33
Seed 5	5.85	-4.27	6.46	-5.7	4.38	-5.37	5.48	-6.31
Seed 6	4.34	-5.57	7.5	-5.98	5.9	-4.69	5.89	-5.23
Seed 7	6.09	-4.17	6.37	-5.69	4.37	-5.22	5.42	-6.22
Seed 8	6.12	-4.13	6.31	-5.71	4.32	-5.34	5.36	-6.4
Seed 9	4.35	-5.51	7.36	-6.04	6.37	-4.62	5.8	-5.24
Seed 10	6.92	-3.19	5.5	-5.71	2.59	-5.2	5.64	-6.02
Seed 11	6.59	-4.08	6.3	-5.67	4.37	-5.16	5.25	-6.33
Seed 12	6.93	-4.08	6.29	-5.68	4.35	-5.28	5.26	-6.44
Seed 13	6.15	-2.65	4.77	-5.77	7.0	-5.42	3.9	-7.95
Seed 14	7.12	-4.08	6.24	-5.74	4.35	-5.44	5.66	-6.23
Seed 15	6.86	-4.12	6.3	-5.72	4.34	-5.56	5.92	-6.07
Seed 16	5.8	-4.25	6.57	-5.57	4.33	-5.25	5.51	-6.17
Seed 17	5.86	-4.21	6.41	-5.69	4.36	-5.4	5.55	-6.27
Seed 18	4.93	-6.7	7.46	-7.14	6.06	-3.38	4.42	-5.38
Seed 19	6.25	-4.06	6.35	-5.64	4.31	-5.16	5.44	-6.58
Seed 20	7.03	-4.08	6.27	-5.71	4.36	-5.4	5.63	-6.19

Table 4. Our reaction rate fits for Scheme 4 for all seeds. Note that parameters are not sorted according to dissociation constants, therefore parameters indicated as being for the C lobe may be representative of the N lobe.

Seed #	k_5	K_{D_5}	k_7	K_{D_7}	k_1	K_{D_1}	k_3	K_{D_3}
Seed 1	5.83	-2.82	7.51	-6.48	4.45	-5.21	4.06	-5.71
Seed 2	6.29	-2.69	7.19	-6.63	4.37	-5.1	4.09	-5.74
Seed 3	6.68	-3.05	7.03	-6.19	4.47	-5.19	4.07	-5.79
Seed 4	5.81	-2.51	7.45	-6.75	4.34	-5.18	3.99	-5.6
Seed 5	6.79	-2.44	7.34	-6.84	3.9	-5.17	5.62	-5.48
Seed 6	4.71	-5.9	5.89	-4.88	5.56	-4.93	4.2	-5.87
Seed 7	5.85	-2.69	7.29	-6.59	4.37	-5.13	4.03	-5.64
Seed 8	6.52	-2.63	7.26	-6.59	4.35	-5.07	4.02	-5.64
Seed 9	3.91	-5.16	5.47	-5.56	6.67	-2.38	7.4	-6.92
Seed 10	6.81	-2.29	7.53	-6.98	3.88	-5.23	5.4	-5.51
Seed 11	5.65	-3.0	7.1	-6.2	4.44	-5.24	4.04	-5.69
Seed 12	6.86	-2.26	7.63	-7.0	3.91	-5.35	5.26	-5.52
Seed 13	5.62	-2.93	7.52	-6.27	4.44	-5.11	4.02	-5.67
Seed 14	6.77	-2.6	7.34	-6.63	4.41	-5.24	4.01	-5.66
Seed 15	6.51	-2.64	7.38	-6.62	4.39	-5.19	3.99	-5.64
Seed 16	5.03	-6.53	5.66	-4.95	3.91	-5.27	2.32	-4.24
Seed 17	4.91	-6.54	5.7	-5.07	3.89	-5.4	1.98	-4.13
Seed 18	4.01	-5.6	7.51	-4.44	5.11	-4.4	2.42	-5.27
Seed 19	5.43	-1.77	8.53	-7.41	4.36	-5.24	3.94	-5.56
Seed 20	5.0	-6.37	5.81	-4.94	3.95	-5.44	2.29	-4.2

Table 5. Our reaction rate fits for Scheme 5 for all seeds. Note that parameters are not sorted according to dissociation constants, therefore parameters indicated as being for the C lobe may be more representative of the N lobe.

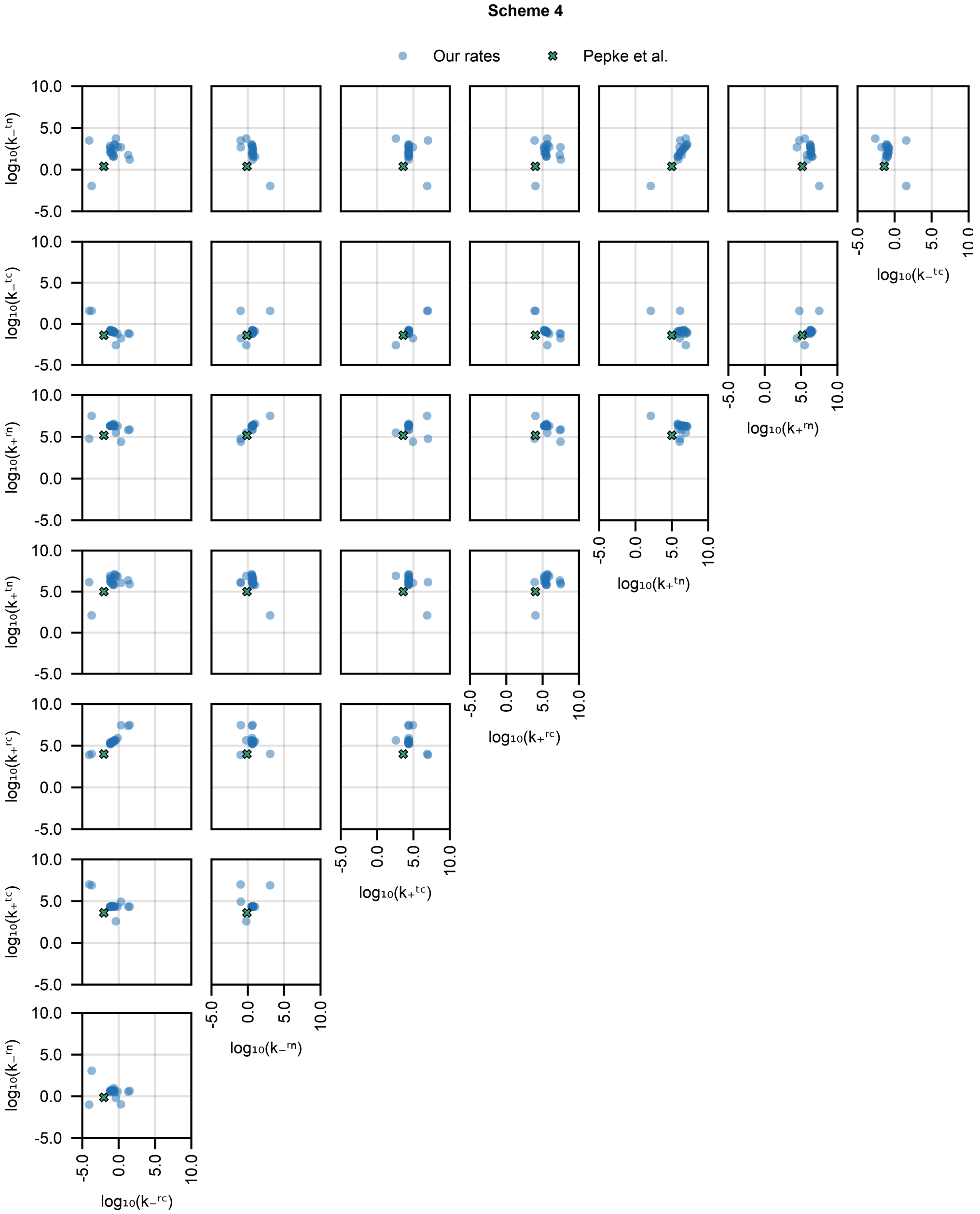

Fig 4. Pair plots for our reaction rate constants for Scheme 4 (blue dots) for all random seeds, along with the published reaction rate constants from Pepke et al. [4].

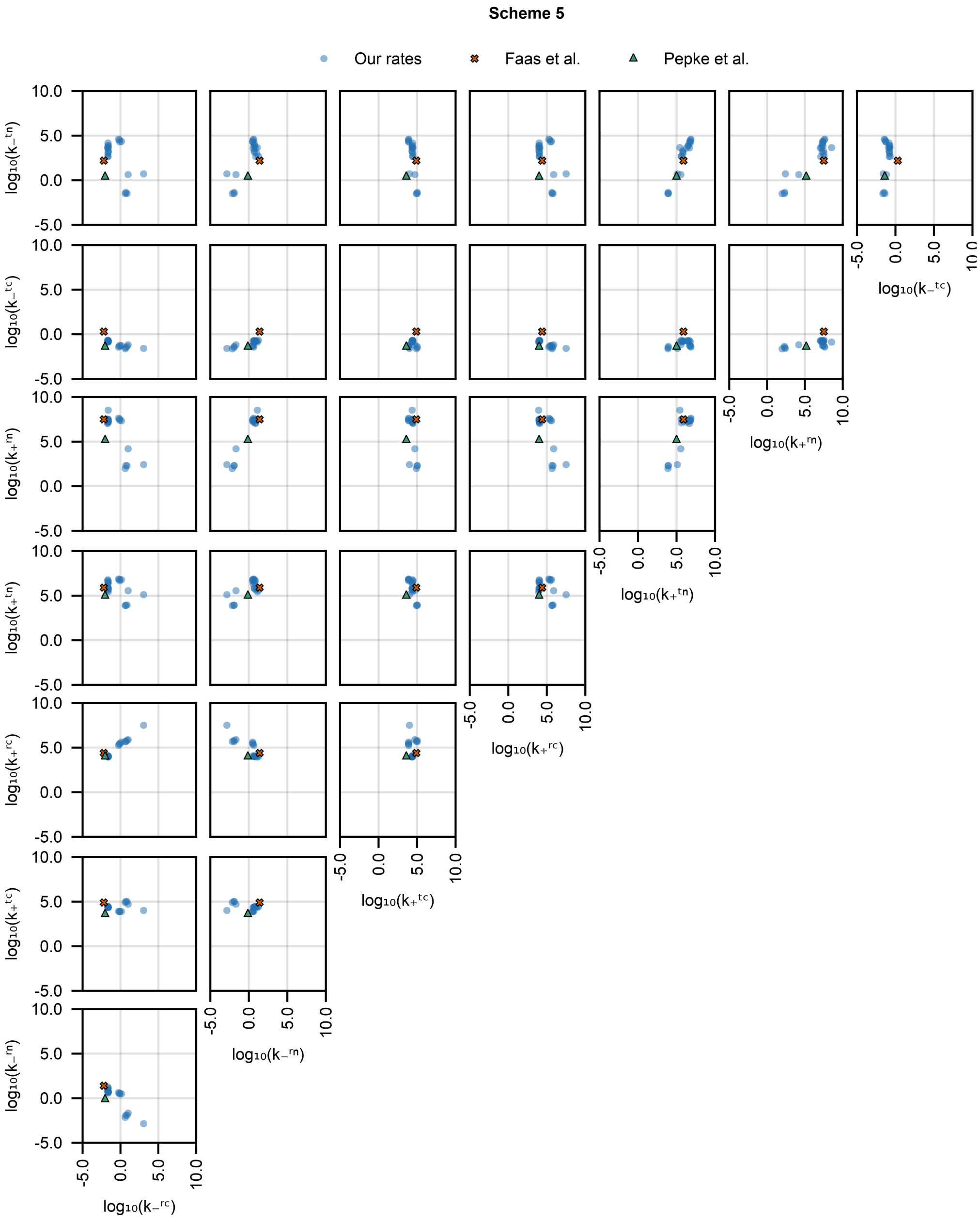

Fig 5. Pair plots for our reaction rate constants for Scheme 5 (blue dots) for all random seeds, along with two sets of published reaction rate constants orange crosses (Faas et al. [5]) and green triangles (Pepke et al. [4]).

Seed #	$\mathbf{k^n_{01}}$	$\mathbf{k^n_{02}}$	$\mathbf{k^n_{13}}$	$\mathbf{k^n_{23}}$	$\mathbf{K^n_{D_{01}}}$	$\mathbf{K^n_{D_{13}}}$	$\mathbf{K^n_{D_{02}}}$	$\mathbf{k^c_{01}}$	$\mathbf{k^c_{02}}$	$\mathbf{k^c_{13}}$	$\mathbf{k^c_{23}}$	$\mathbf{K^c_{D_{01}}}$	$\mathbf{K^c_{D_{13}}}$	$\mathbf{K^c_{D_{02}}}$
1	3.29	6.26	5.25	7.63	-3.34	-5.91	-2.92	4.12	4.23	1.89	5.27	-4.46	-5.41	-6.32
2	6.55	4.37	7.07	5.78	-3.06	-6.2	-3.16	3.99	4.22	2.03	5.32	-4.34	-5.36	-6.43
3	3.72	6.73	3.39	7.3	-4.4	-4.78	-2.92	4.0	4.52	1.54	4.23	-5.45	-4.9	-5.24
4	5.33	6.21	5.86	7.47	-2.77	-6.47	-2.77	4.05	4.2	1.89	5.24	-4.44	-5.34	-6.3
5	6.81	3.86	7.23	3.47	-2.93	-6.36	-4.23	4.22	6.49	3.53	6.06	-5.32	-2.59	-5.23
6	3.9	3.87	5.32	5.47	-5.23	-5.42	-4.98	5.44	6.92	3.57	7.47	-2.39	-2.53	-6.93
7	3.42	6.66	5.23	7.35	-4.0	-5.27	-2.82	4.33	6.12	3.68	6.16	-5.4	-2.4	-5.21
8	4.29	6.65	5.42	7.24	-3.29	-5.92	-2.95	4.2	4.04	5.3	2.27	-5.31	-4.37	-5.38
9	3.63	6.74	5.12	7.33	-3.67	-5.58	-2.9	4.08	4.24	2.21	5.32	-4.41	-5.36	-6.39
10	3.13	6.42	3.33	7.31	-4.25	-4.95	-2.99	4.07	4.22	1.99	5.42	-4.36	-5.39	-6.32
11	5.97	5.36	7.28	5.72	-3.11	-6.06	-2.77	4.16	4.26	1.98	5.34	-4.49	-5.4	-6.37
12	5.32	6.69	5.87	7.46	-2.95	-6.23	-2.94	4.16	4.27	2.12	5.36	-4.42	-5.47	-6.47
13	5.25	6.61	5.98	7.26	-3.06	-6.18	-3.02	4.3	6.43	3.6	6.09	-5.34	-2.49	-5.26
14	4.16	4.26	2.53	5.34	-4.42	-6.42	-5.33	5.43	6.94	3.57	7.51	-2.59	-2.78	-6.63
15	3.07	6.68	5.2	7.56	-3.97	-5.31	-2.84	4.14	4.24	1.98	5.28	-4.4	-5.37	-6.39
16	4.03	4.18	2.67	5.34	-4.38	-6.27	-5.31	5.66	5.59	7.31	5.04	-3.26	-2.53	-5.96
17	3.24	6.68	3.51	7.39	-4.31	-4.99	-2.72	4.28	6.49	3.62	6.25	-5.3	-2.4	-5.25
18	5.67	5.4	7.33	5.61	-3.12	-6.12	-2.69	4.39	3.92	4.11	1.88	-5.12	-5.24	-5.54
19	3.8	4.16	4.61	6.49	-5.82	-4.01	-4.79	5.41	5.45	0.33	5.37	-4.62	-1.72	-5.11
20	4.17	4.28	2.52	5.36	-4.48	-6.41	-5.36	5.41	6.79	3.57	7.4	-2.72	-2.93	-6.5

Table 6. Our reaction rate fits for Scheme 6 for all seeds. Note that parameters are not sorted according to dissociation constants, therefore parameters indicated as being for the C lobe may be more representative of the N lobe. Also, note that $K^c_{D_{23}}$ is absent as it is not a free parameter, but expressed via other dissociation constants due to microscopic reversibility constraints – $K_{D_{23}} = \frac{K_{D_{01}} K_{D_{13}}}{K_{D_{02}}}$.

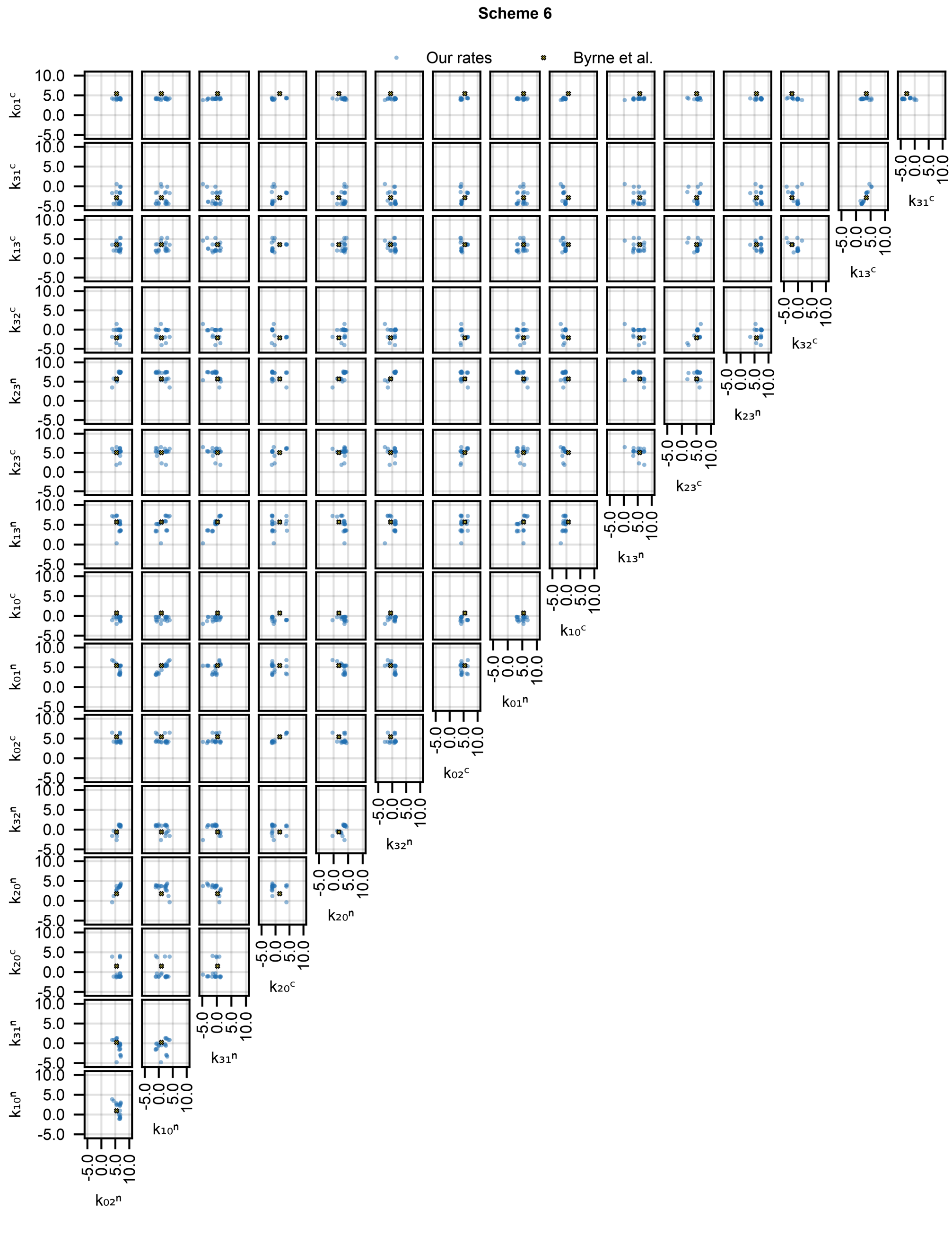

Fig 6. Pair plots for our reaction rate constants for Scheme 6 (blue dots) for all random seeds, along with the published reaction rate constants from Byrne et al. [6].
