# Supplementary material for: Fitting and comparison of calcium-calmodulin kinetic schemes to a common data set using non-linear mixed effects modelling
Source: PLoS One. 2025 Feb 7;20(2):e0318646. doi: 10.1371/journal.pone.0318646 (PMC11805441; doi:10.1371/journal.pone.0318646)
Supplement: Attachment — Submitted filename: Respone_to_reviewers.pdf [file pone.0318646.s007.pdf]

School of informatics

THE UNIVERSITY OF EDINBURGH

11 December 2024

INSTITUTE *for* ADAPTIVE *and* NEURAL COMPUTATION

SCHOOL *of* INFORMATICS
The University of Edinburgh
The Informatics Forum
10 Crichton Street
Edinburgh EH8 9AB

Telephone 0131 650 3099
Fax 0131 650 6899
E-mail D.Linkevicius@sms.ed.ac.uk

Pan Li
Academic Editor
PLoS ONE

Manuscript submission: "Fitting and comparison of calcium-calmodulin kinetic schemes to a common data set using non-linear mixed effects modelling"
Domas Linkevicius, Angus Chadwick, Guido Faas, Melanie I. Stefan & David C. Sterratt

Dear Dr Li,

We are grateful to the Reviewers for their insightful comments and observations regarding our manuscript. We will first address what we see as the major points of discussion, followed by more minor points.

Reviewer #1 wrote: "In my opinion, the manuscript lacks novelty, since, there is no new modelling scheme that the authors propose, nor do they propose novel schemes for data fitting."

It is true that the current manuscript does not contain a novel calmodulin scheme or a new fitting technique. However, creation of novel models or fitting techniques is not the only possible source of novel scientific insight. The calmodulin model comparison work contained in this manuscript has not been done before and, therefore, leads to new insights: the needlessness of new calmodulin modeling schemes given the current available data, the level of detail necessary to model calcium-calmodulin binding. Furthermore, the work highlights the potential pitfalls of using calmodulin schemes that are too simple to model calcium-calmodulin binding, which calls for a reassessment of existing work. Therefore, despite the manuscript not containing new modeling schemes or parameter estimation methods, it is still original and insightful.

Reviewer #1 also wrote: "You have mentioned in page 12 as well as in the Discussion section that the parameters that you have estimated fit the data of Faas et.al. [5] and Shifman et. al. [11] better than the parameters in existing literature in many cases as also seen from the RMSE values in Table 1. I think this is obvious, because the published rate constants may have been derived using other sets of data."

The fact that our estimates result in better fits than the previously published rate constants is obvious only at a first glance. Upon deeper reflection it brings up a number of important points, especially taking into account the magnitude of the difference in some comparisons. Even though different published rate constants have been derived using different sets of data, there still ought to be broad agreement in model predictions if the data sets are broadly consistent. If the model predictions are not consistent (especially qualitatively, rather than quantitatively), then either

the different data sets are contradictory or some of the models are not powerful enough to fit certain data sets (or both). Our comparisons revealed a number of cases where the differences are qualitative, rather than quantitative, namely Schemes 1–3 and parameters from Pepke et al. show significant qualitative differences from our fits. This, however, is not the case for the Faas et al. or the Byrne et al. parameters, where the differences are much smaller and keep to the same qualitative trends as our fits. Therefore, yes, it is unsurprising that our fits result in lower errors on the data we use to fit them than other models which were tuned to different data sets, but the size of errors is, in some cases, surprising.

Reviewer #1 also pointed out that the parameter estimation procedure could be explained better. We have added additional explanations, expanding and deepening the level of explanation. We have also addressed the following list of more minor remarks (following the numbering in the review):

1. In page 6, 7, one of the references did not appear; this has been corrected.
2. The explanation of why the nine states described in the beginning of the second paragraph of page 7 can be reduced to 5 states was found to be lacking. We clarified this point and used more precise language, making it clear that this is not a conceptual reduction of the model, but simply of the states being recorded, the underlying kinetic scheme is the same.
3. The term "rates" was often used instead of "rate constants"; this has been corrected.
4. We have fixed the spelling error pointed out by the Reviewer.
5. This point about the argument about our parameters fitting better than previous ones because previous parameters have been tuned to other data has been addressed in the paragraph above.
6. The Reviewer pointed out that it is not clear how the model in equation (5) represents frequency/signals. We have added additional description to clarify that calcium injections are a part of the differential equation system and can be used to model calcium signals of different frequency or amplitude.

Moving on to the comments provided by Reviewer #2, the Reviewer wrote: "The fact that Equation 2 fits better than Equation 1 means that the strength of the laser pulse is completely random and cannot be controlled by specifying a PCD to a relevant degree. I think this should be clarified."

We appreciate why the reviewer would draw this conclusion. With our approach, we do not claim that uncaging is completely independent of PCD; rather we use a single equation to capture both uncertainty in estimating the PCD, as well as other sources of variance. The amount of variance captured by the specified PCD value and finer points of calcium uncaging modeling are left for future research. We have included a statement to this effect in the revised version.

We addressed the feedback by Reviewer #2 concerning the usage of terminology and adjusted the relevant parts to follow Duchesne et al. (2021). We clarified and reduced the abuse of notation for the NLME parameters. We changed the symbol η to a more neutral symbol x to show that it is simply a description of a function. We clarified that the only place we used random effects is the calcium uncaging fraction. We clarified how the random effect prior distribution parameters were constrained. We expanded the section on model comparison, specifying that Laplace approximated marginal likelihood was used in AIC computations, described how and why we used Cohen's d. We also addressed the further more minor notes provided by Reviewer #2, correcting the missing references, parentheses and other grammatical errors.

We again thank the Reviewers for their time, insightful feedback and contributing to the scientific community. We hope that our clarifications and revisions address their feedback satisfactorily.

Many thanks for your consideration,

Domas Linkevicius
